# New species-specific quantitative PCR assays for *Liriomyza* leafminers: supporting biosecurity and in-field identification

**DOI:** 10.1093/jee/toag119

**Published:** 2026-05-11

**Authors:** Anthony R J van Rooyen, Andrew R Weeks, Elia I Pirtle, Peter M Ridland, John Duff, Mallik B Malipatil, John G Black, Haylo K Roberts, Helen Spafford, Kim Saligari, Holly Bridge-Cattermole, Carolyn Church, Olivia L Reynolds, Paul A Umina

**Affiliations:** Cesar Australia, Brunswick, Victoria, Australia; Cesar Australia, Brunswick, Victoria, Australia; Cesar Australia, Brunswick, Victoria, Australia; School of BioSciences, The University of Melbourne, Parkville, Victoria, Australia; Department of Primary Industries, Gatton Research Facility, Gatton, Queensland, Australia; Museums Victoria, Carlton, Victoria, Australia; Cesar Australia, Brunswick, Victoria, Australia; Cesar Australia, Brunswick, Victoria, Australia; Department of Primary Industries and Regional Development, Frank Wise Institute of Tropical Agriculture, Kununurra, Western Australia, Australia; Department of Primary Industries and Regional Development, Frank Wise Institute of Tropical Agriculture, Kununurra, Western Australia, Australia; Department of Primary Industries and Regional Development, Frank Wise Institute of Tropical Agriculture, Kununurra, Western Australia, Australia; Department of Primary Industries, Gatton Research Facility, Gatton, Queensland, Australia; Cesar Australia, Brunswick, Victoria, Australia; School of Agricultural, Environmental and Veterinary Sciences, Charles Sturt University, Wagga Wagga, New South Wales, Australia; Cesar Australia, Brunswick, Victoria, Australia; School of BioSciences, The University of Melbourne, Parkville, Victoria, Australia

**Keywords:** agromyzid, invasive species, assay development, environmental DNA, in-field detection

## Abstract

The Agromyzidae are a diverse family of small flies whose larvae mine plant tissues, often with a high degree of host specificity. However, several species of *Liriomyza—*including the vegetable leafminer (*Liriomyza sativae* Blanchard), pea leafminer (*Liriomyza huidobrensis* [Blanchard]), American serpentine leafminer (*Liriomyza trifolii* [Burgess]) and tomato leafminer (*Liriomyza bryoniae* [Kaltenbach]) - are highly polyphagous and pose significant biosecurity risks to Australian agriculture. The stone leek leafminer (*Liriomyza chinensis* [Kato]), though host-specific to *Allium* spp., is also a concern. Effective surveillance and diagnosis are challenging due to the flies’ small size, rapid life cycles, and morphological similarities. While previous quantitative PCR (qPCR) assays have enabled detection of *L. sativae*, *L. trifolii*, and *L. huidobrensis*, and the non-pest species *Liriomyza brassicae* (Riley) (cabbage leafminer), we extend this capability by developing species-specific assays for *L. bryoniae* and *L. chinensis*. The new assays were highly specific across 16 agromyzid species collected globally and demonstrated strong sensitivity. A revised assay for *L. huidobrensis*, targeting the mitochondrial ND5 region, eliminated cross-reactivity observed with the original qPCR assay. We also validated a simplified DNA extraction method suitable for field use and demonstrated successful in-field diagnosis using the portable Franklin^TM^ three9 real-time PCR themocycler. These assays have already proven valuable during post-incursion surveys and general surveillance efforts. Together, they provide a flexible and rapid diagnostic toolkit to support the early detection and management of *Liriomyza* pests in Australia and elsewhere.

## Introduction

*Liriomyza* flies (Diptera: Agromyzidae) represent a major risk to Australia’s agricultural sector, particularly to the vegetable, potato, melon and nursery industries (Jovicich 2009). Approximately 3,000 species of Agromyzidae have been described worldwide (Lonsdale et al. 2023), all of which are morphologically similar and difficult to distinguish (Scheffer et al. 2007). While most agromyzid species are host-specific, a few are highly polyphagous and have emerged as globally important agricultural pests (Spencer 1973). Crop damage is primarily caused by larval feeding between the upper and lower surfaces of leaves, which reduces photosynthetic capacity and diminishes the yield and marketability of affected plants.

*Liriomyza sativae* Blanchard attacks a broad range of vegetable and flowering crops (Spencer 1973, 1990) and is found in more than 75 countries across North America, South America, Europe, Asia, and Africa (Ridland et al. 2020). In 2008, *L. sativae* was detected for the first time in the Torres Strait, and then on the Australian mainland near Seisia in the Northern Peninsula Area of Cape York (Queensland) in 2015 (Blacket et al. 2015, IPPC 2017). Other significant *Liriomyza* pest species include *Liriomyza trifolii* (Burgess), *Liriomyza huidobrensis* (Blanchard), *Liriomyza bryoniae* (Kaltenbach) and *Liriomyza chinensis* (Kato) (Spencer 1973, Parrella and Keil 1984, Spencer 1990, Lonsdale et al. 2023). All 4 species are important pests globally and, like *L. sativae*, are recognized as biosecurity threats in Australia (Ridland et al. 2020, Lonsdale et al. 2023). In 2020, *L. huidobrensis* was first detected in the Sydney region and later found at multiple locations in southern and central New South Wales, as well southern Queensland (IPPC 2021a, Mulholland et al. 2022). In 2022, it was also detected on celery crops near Werribee, Victoria. *Liriomyza huidobrensis* has been recorded from over 250 plant species across 50 plant families (Spencer 1973, Weintraub et al. 2017). Important agricultural hosts include a wide range of vegetables, potatoes, pulses, oilseeds, herbs, cereals, and some pasture species (Spencer 1973, 1990, Weintraub et al. 2017). *Liriomyza trifolii* has also recently established in Australia. It was first detected in Kununurra (Western Australia) and within the Torres Strait in 2021, and subsequently in Broome (Western Australia), Darwin and Katherine (Northern Territory), and near Bamaga in the Northern Peninsula Area of Cape York (IPPC 2021b). Important agricultural hosts include sunflower, safflower, lettuce, celery, cucumber, melons, onion, potato, tomato, eggplant, legume vegetables, and pulse crops, as well as a wide range of ornamental plants (Lonsdale et al. 2023). In 2025, *L. trifolii* was also recorded in the Lockyer Valley (Queensland) on cover crops (Sunn hemp and cowpea), and on eggplants and capsicums in protected cropping structures (AusVeg 2025). Predictive modelling by Maino et al. (2023) has identified many regions in Australia with high ecological suitability for *L. sativae, L. huidobrensis* and *L. trifolii*, suggesting their ranges are likely to expand significantly and they have the potential to cause considerable economic impact to Australia’s horticultural industries.

*Liriomyza bryoniae* and *L. chinensis* are currently absent from Australia but remain key biosecurity threats*. Liriomyza bryoniae* is native to North–Western Europe but is also widespread in Asia and parts of Africa (Lonsdale et al. 2023). It has been recorded from 16 plant families (Spencer 1973) and is an important pest of tomatoes, cabbage, cucurbits, eggplants, potatoes, lettuce, and beans. *Liriomyza chinensis* is a pest of *Allium* crops in many regions, especially in East Asia. Notably, *L. bryoniae* and *L. chinensis* are widespread in China (Lonsdale et al. 2023), one of Australia’s major trading partners.

The cryptic nature and wide host ranges of *Liriomyza* pests have made them historically difficult to detect and challenging to identify. Even at the adult stage, *Liriomyza* flies are morphologically very similar (Spencer 1973, Scheffer et al. 2001). Species can be identified on the basis of male genitalia, but this method is time consuming and requires specialist expertise (Malipatil and Ridland 2008). Morphological differentiation of adult females and juvenile stages is particularly difficult and often unreliable. Consequently, molecular DNA-based approaches have been developed to enable more accurate species identification (eg Scheffer et al. 2006, 2014, Blacket et al. 2015, Zhu et al. 2023). Sooda et al. (2017) developed a quantitative PCR (qPCR) assay with species-specific primers and probes for detecting *L. sativae, L. trifolii*, and *L. huidobrensis*, demonstrating high specificity. Building on this, Pirtle et al. (2021) adapted the assay for environmental DNA (eDNA) detection of *L. sativae* from “empty” leaf mines. This approach was expanded to include *Liriomyza brassicae* (Riley), an introduced species in Australia that is widespread but not regarded as an important agricultural pest (Xu et al. 2021). The eDNA assay is valuable because it targets residual DNA from leaf mines, which is simple to collect and persists longer in the environment than the adult flies. Consequently, the method extends the detection window in the field, improves surveillance sensitivity, and is likely applicable to other *Liriomyza* species.

In this study, we extend the qPCR assay from Sooda et al. (2017) and Pirtle et al. (2021) by designing species-specific assays for *L. bryoniae* and *L. chinensis.* We validated the specificity of our assays against a broad range of target and non-target species collected both within Australia and internationally. In addition, we refined the *L. huidobrensis* primers and probes from Sooda et al. (2017) to resolve cross-amplification with *L. bryoniae*. Finally, we evaluated the utility of these new assays, in combination with a simple DNA extraction method, as a rapid field test using the portable Franklin^TM^ real-time PCR thermocycler, applying it to larval and leaf mine samples.

## Materials and Methods

### Sample Collection and Identification

More than 300 insect samples were collected from across Australia for this study. These comprised 4 of the 6 target *Liriomyza* species (*L. sativae, L. huidobrensis, L. trifolii*, and *L. brassicae)*, as well as 8 non-target leafmining species (Table 1). Target specimens were collected across a variety of developmental stages (larvae, pupae, adults, and empty leaf mines) and from a diverse range of host plant species. All samples were preserved in 80% ethanol upon collection and stored at −20 °C until further analysis. Adult flies were predominantly obtained by rearing them from field-collected larvae or pupae, whereas larval, pupal, and empty leaf mine samples were collected directly from infested host plant material and preserved immediately.

**Table 1.** Summary of specimens collected in Australia and average *Cq* values (and range) from species-specific qPCR assays used in this study

Species	Development stage	State/Territory	Plant hosts	No. samples	*Cq* values						
					*Liriomyza sativae* ^a^	*Liriomyza trifolii* ^a^	*Liriomyza huidobrensis* ^a^	*Liriomyza huidobrensis* ^b^	*Liriomyza brassicae* ^c^	*Liriomyza bryoniae* ^b^	*Liriomyza chinensis* ^b^
***Liriomyza sativae***	Larvae, pupae	QLD	Cucurbitaceae (family), *Macroptilium atropurpureum, Solanum lycopersicum*	4	20 (19 to 21)						
***Liriomyza trifolii***	Flies, leaf mines	NT, QLD, WA	*Amaranthus* sp., *Beta vulgaris, Brassica rapa* subsp. *chinensis*, *Brassica rapa* subsp. *nipposinica*, *Carthamus tinctorius, Citrullus lanatus, Cucumis sativus, Cyanthillium cinereum, Gossypium* sp., *Helianthus annuus, Lactuca sativa, Phaseolus* sp., *Phaseolus vulgaris, Physalis angulata, Pisum sativum, Tagetes* sp., *Zinnia elegans*	112		21 (16 to 39)					
***Liriomyza huidobrensis***	Flies, larvae, leaf mines	QLD	*Allium cepa, Amaranthus* sp., *Apium graveolens, Beta vulgaris* subsp. *vulgaris*, *Brassica oleracea* var. *capitata*, *Brassica oleracea* var. *italica*, *Brassica oleracea* var. *sabellica*, *Brassica rapa* subsp. *chinensis*, *Brassica rapa* subsp. *pekinensis*, *Capsella bursa-pastoris, Chenopodium album, Conyza* sp., *Datura ferox, Fallopia convolvulus, Galinsoga parviflora, Hibiscus trionum, Lactuca sativa, Lamium* sp., *Malva parviflora, Polygonum erectum, Senecio madagascariensis, Solanum lycopersicum, Solanum tuberosum, Sonchus oleraceus, Stellaria media, Trifolium* sp., *Urtica dioica*	121			20 (17 to 37)	21 (17 to 38)			
***Liriomyza brassicae***	Flies, leaf mines	QLD, Vic.	*Brassica juncea, Brassica oleracea* var. *capitata*, *Brassica oleracea* var. *italica*, *Brassica oleracea* var. *sabellica*, *Brassica* sp., *Tropaeolum majus*	24					21 (19 to 34)		
***Liriomyza chenopodii***	Flies	Vic.	*Beta vulgaris, Tropaeolum majus* ^d^	4							
***Calycomyza humeralis***	Flies	QLD	*Conyza* sp.	9							
***Cerodontha milleri***	Flies	Vic.	*Hordeum vulgare*	4							
***Ophiomyia alysicarpi***	Flies	QLD	*Alysicarpus* sp.	4							
***Ophiomyia solanicola***	Flies	QLD	*Solanum melongena*	4							
***Phytomyza plantaginis***	Flies	Vic.	*Plantago lanceolata*	3							
***Phytomyza syngenesiae***	Flies, pupae	QLD, Vic.	*Sonchus asper, Sonchus oleraceus*	15							
***Scaptomyza flava***	Larvae	Vic.	*Brassica oleracea* var. *capitata*, *Eruca sativa, Spinacia oleracea, Brassica rapa* var. *nipposinica*	4							

a From assay developed by Sooda et al. (2017).

b From assays developed in this study.

c From assay developed by Pirtle et al. (2021).

d We do not consider *Tropaeolum majus* to be a new plant host of *L. chenopodii*.

QLD, Queensland; NT, Northern Territory; WA, Western Australia; Vic., Victoria.

To supplement our Australian collection, we sourced specimens from international collaborators. A total of 56 international samples were included in our study, encompassing all 6 target species (*L. sativae, L. huidobrensis, L. trifolii, L. brassicae, L. bryoniae*, and *L. chinensis)*, as well as 2 additional non-target species (*Liriomyza katoi* Sasakawa, and *Liriomyza yasumatsui* Sasakawa). These samples were obtained from a broad geographic range, including Fiji, Indonesia, Kenya, Netherlands, Solomon Islands, Timor Leste, Vietnam, and the United States of America (Table 2). This diverse set of specimens ensured robust validation of our assays across a wide phylogenetic and geographic spectrum.

**Table 2.** Summary of specimens collected from countries outside of Australia and average *Cq* values (and range) from species-specific qPCR assays used in this study

Species	Development stage	Country	No. samples	*Cq* values						
				*Liriomyza sativae* ^a^	*Liriomyza trifolii* ^a^	*Liriomyza huidobrensis* ^a^	*Liriomyza huidobrensis* ^b^	*Liriomyza brassicae* ^c^	*Liriomyza bryoniae* ^b^	*Liriomyza chinensis* ^b^
***Liriomyza sativae***	Flies, larvae	Indonesia, Kenya, Timor-Leste, USA, Vietnam	17	22 (18 to 31)						
***Liriomyza trifolii***	Flies, larvae	Fiji, Indonesia, Kenya, Solomon Islands, Timor-Leste, USA	18		21 (18 to 35)					
***Liriomyza huidobrensis***	Flies	Indonesia, Kenya	9			21 (18 to 28)	23 (18 to 27)			
***Liriomyza brassicae***	Flies	Indonesia, Timor-Leste	4					22 (19 to 32)		
***Liriomyza bryoniae***	Flies	Netherlands	3			19 (18 to 20)			18 (17 to 19)	
***Liriomyza chinensis***	Flies	Indonesia	3							19 (18 to 19)
***Liriomyza katoi***	Flies	Indonesia	1							
***Liriomyza yasumatsui***	Flies	Indonesia	1							

a From assay developed by Sooda et al. (2017).

b From assays developed in this study.

c From assay developed by Pirtle et al. (2021).

Prior to molecular analysis, flies were identified using morphological keys, primarily based on the genitalia of adult males (Bock 1977, 1982, Spencer 1977, 1990).

### DNA Extraction from Insect and Leaf Mine Samples

DNA was extracted from insect and leaf mine samples using different protocols depending on the sample origin and intended downstream application.

For initial specificity testing, including all overseas specimens, genomic DNA (gDNA) was extracted from individual insect samples using the Qiagen DNeasy Blood & Tissue Kit (Qiagen, Hilden, Germany), following the manufacturer’s standard spin-column protocol. Extractions were performed on whole adult flies, pupae or larvae, which were crushed using a sterile pipette tip prior to lysis to ensure efficient cell disruption and DNA release.

For samples collected in southern Queensland and Western Australia, DNA was extracted from adult flies, larvae, and empty leaf mine samples using a modified Chelex 100 extraction protocol, as described by Pirtle et al. (2021). This approach was selected due to its speed, simplicity, and cost-effectiveness, particularly in high-throughput and field-relevant contexts.

For field-deployable qPCR assays using the Franklin^TM^ real-time PCR thermocycler, a simplified extraction method was used to facilitate *in situ* sample processing. For leaf mine samples, a ∼10 × 2 mm section of mined leaf tissue was excised and crushed in 100 µl of Qiagen Buffer AE using a sterile pipette tip. For larval samples, individual larvae were carefully removed from their mines and similarly crushed in 100 µl of Qiagen Buffer AE.

All extracted DNA samples, whether from Chelex, spin-column, or using field protocols, were stored at −20 °C until assayed. This combination of standard and rapid extraction protocols allowed us to evaluate assay performance across both laboratory and field conditions, and to test compatibility with high- and low-quality DNA inputs.

### qPCR Assay Design

Multi-species real-time PCR assays targeting *L. sativae, L. trifolii and L. huidobrensis* have previously been developed by Sooda et al. (2017). The *L. sativae* assay was later applied to leaf mine samples as part of the eDNA approach developed by Pirtle et al. (2021), which also encompassed *L. brassicae*. In initial *in silico* analyses, we observed that the *L. huidobrensis* assay primers and probes showed sequence matches with *L. bryoniae*, suggesting cross-reactivity. To assess this, we screened the original assays for specificity against a panel of non-target *Liriomyza* species collected from various countries (see below).

To develop new species-specific assays, we downloaded complete mitochondrial genome sequences from GenBank (www.ncbi.nlm.nih.gov) for *L. huidobrensis, L. bryoniae, L. chinensis* and all relevant non-target species. These sequences were aligned using Geneious v10.2.5 (https://www.geneious.com) and unique regions were identified for each target species. Candidate primer and probe sets were designed using the custom TaqMan^TM^ Assay Design tool (https://www.thermofisher.com/order/custom-genomic-products/tools/cadt/) and their specificity assessed *in silico* using Primer-Blast (https://www.ncbi.nlm.nih.gov/tools/ primer-blast/). No non-target cross-amplifications were predicted.

Species-specific TaqMan^TM^ copy number assays were ordered from Thermo Fisher Scientific (Waltham, MA, United States), labeled with FAM fluorophores. Once validated individually on all target and non-target gDNA samples (see below), assays were transitioned to the PrimeTime^TM^ qPCR method (Integrated DNA Technologies) using HEX and Cy5 fluorophores (Table 3), enabling multiplexing in 2 reactions and detection across 3 fluorophore channels.

**Table 3.** Primers and labelled probe sequences targeting mitochondrial gene regions for the 6 target *Liriomyza* species

Species	Gene region	Amplicon size (bp)	Primer/probe sequences
***Liriomyza sativae* ^a^**	CO1	109	Primer 1 5′- ACCCCCTGCTTTAACTCTTTT -3′ Primer 2 5′- AGCACCACCATGTGCAATAA -3′ Probe FAM- CAGTATAGTAGAAAATGGGGCTGGGA -NFQ
***Liriomyza trifolii* ^a^**	CO1	66	Primer 1 5′- CGGAGCTGGTACAGGATGA -3′ Primer 2 5′- GAAGCTCCACCATGTGCAATA -3′ Probe FAM- CCGTTTACCCTCCCCTTTCCTCA -NFQ
***Liriomyza huidobrensis* ^a^**	CO1	112	Primer 1 5′- CCTCCAGCTCTTACCCTTCTAC -3′ Primer 2 5′- CTGAAGCTCCTCCATGAGCAA -3′ Probe FAM- AAGAAGTATAGTTGAAAACGGAGCTGGGA -NFQ
***Liriomyza huidobrensis* ^b^**	ND5	132	Primer 1 5′- ATAAACTACCCATTCAGCTATCTAAT -3′ Primer 2 5′- CATGACTTCCAGCAGCTAT -3′ Probe FAM- CCCTGCCGTAACCAA -NFQ
***Liriomyza brassicae* ^c^**	CO1	63	Primer 1 5′- GCCGGAACAGGATGAACAGTTTAT -3′ Primer 2 5′- AGATGCCCCACCGTGAG -3′ Probe FAM- CCCCTCTCTTCTATTATTG -NFQ
***Liriomyza bryoniae* ^b^**	ND5	129	Primer 1 5′- AAAAACTCCCTAATTCTCTATCCAAT -3′ Primer 2 5′- GGCTTCCTGCTGCAATAGC -3′ Probe FAM- CCAGCTGTAACTAAAGTTG -NFQ
***Liriomyza chinensis* ^b^**	CO1	146	Primer 1 5′- CCCAGCACTTACTTTACTTTTATTAAGAAG -3′ Primer 2 5′- TTCCTGCGAGATGTAAAGAGAA -3′ Probe FAM- CTCCATGGGCGATTAC -NFQ

a From assay developed by Sooda et al. (2017).

b From assays developed in this study.

c From assay developed by Pirtle et al. (2021).

### qPCR Assays—Roche LightCycler

All TaqMan and PrimeTime qPCR assays were run on a Roche LightCycler 480 II system using a 384-well plate format. Each 10 µl reaction contained 5 µl of KAPA Probe Force PCR Master Mix (KAPA Biosystems, Cape Town, South Africa), 0.5 µl of TaqMan or PrimeTime qPCR assay mix, 2.5 µl ddH_2_O, and 2 µl of template DNA. Each sample was run in triplicate.

Each plate included a 10-fold serial dilution of gDNA (ranging from 100,000 to 10 fg) to generate standard curves, as well as no-template controls. Amplification occurred under the following cycling conditions: 3 min at 98 °C, followed by 50 cycles of 10 s at 95 °C and 20 s at 60 °C. The amplification profiles of each PCR product were used to determine the cycle quantification (*Cq*) value using the Absolute Quantification module of the LightCycler 480 II software. A TaqMan Exogenous Internal Positive Control (VIC-labelled probe) was included in each sample to assess PCR inhibition. All qPCRs were performed in a dedicated PCR laboratory, physically separated from the DNA extraction area. Negative controls were included at all stages (DNA extraction and amplification), and no evidence of contamination was detected.

### qPCR Assays—Franklin three9 System

Real-time TaqMan and PrimeTime qPCR assays were conducted using the Franklin^TM^ three9 real-time PCR thermocycler (Biomeme Inc., Philadelphia, PA, USA), a portable 9-well field-ready thermocycler. We initially tested 8 fresh *L. brassicae* larvae excised from leaf mines in a laboratory colony (host plant: *Brassica rapa* subsp. *chinensis*) along with 16 empty leaf mines from the same colony. Each 20 µl reaction contained 10 µl of KAPA Probe Force PCR Master Mix (Merck), 1 µl of TaqMan or PrimeTime qPCR assay mix, 5 µl ddH2O, and 4 µl of template DNA. A no-template negative control was included in each run. Due to the limited well capacity of the Franklin system, the positive control dilution series (ranging from 100,000 to 10 fg of gDNA) was run separately.

Cycling conditions were identical to those used in the LightCycler: 3 min at 98 °C, followed by 50 cycles of 10 s at 95 °C and 20 s at 60 °C. *Cq* values were analyzed using the Biomeme Go software package. PCR inhibition was assessed using the TaqMan Exogenous Internal Positive Control on the LightCycler, and any sample showing signs of inhibition was diluted 10-fold and re-analyzed. Negative controls were included in all extraction and amplification runs, and no contamination was detected. Only one technical replicate was run per assay per sample due to well limitations of the Franklin system.

### Primer Efficiency, Limit of Detection, and Limit of Quantification

Primer efficiency, limit of detection (LOD), and limit of quantification (LOQ) were determined following the curve fitting method of Klymus et al. (2020) in R (version 4.5.1; R Core Team 2025). Serial 10-fold dilutions of gDNA (100,000 to 1 fg) were prepared in Qiagen Buffer AE and quantified using a Qubit 2.0 fluorometer (Invitrogen, Carlsbad, CA, United States). Each dilution was run in ten replicates, and the resulting *Cq* values were used to construct standard curves. Primer efficiency, LOD, and LOQ were estimated by plotting *Cq* values against DNA concentrations and determining the linear slope and the coefficient of determination (*R*^2^) values.

We also assessed the amplification efficiency of the *L. brassicae* assay on the Franklin system using the same gDNA dilution series, allowing direct comparison with the results obtained from the Roche LightCycler.

### Specificity Testing

Each species-specific assay was tested for cross-reactivity using 1 ng of gDNA extracted from 1 to 3 individuals of all 16 species originating from Australia and internationally. Tested species included: *L. sativae*, *L. trifolii*, *L. huidobrensis*, *L. bryoniae*, *L. chinensis*, *L. brassicae*, *L. katoi*, *L. yasumatsui*, *Calcomyza humeralis* (Roser), *Cerodontha milleri* Spencer, *Liriomyza chenopodii* (Watt), *Ophiomyia alysicarpi* (Bezzi), *Ophiomyia solanicola* (Spencer), *Phytomyza plantaginis* Goureau, *Phytomyza syngenesiae* (Hardy) and *Scaptomyza flava* (Fallén). All qPCR assays were performed under standard conditions as described above, including appropriate negative controls. Specificity testing was repeated following conversion to the PrimeTime format using different fluorophores.

To confirm species identity and validate our reference panel, all target and non-target species were amplified by PCR using the CO1 barcoding primers LCO1490 and HCO2198 (Folmer et al. 1994), tagged with M13 tail sequences. Amplicons were then Sanger sequenced bidirectionally using M13 primers on an ABI 3730xl system (Macrogen, Seoul, Korea) and compared with reference sequences on GenBank and the BOLD database to confirm species haplotype authenticity and assay specificity. A total of 98 individuals were sequenced: 42 Australian samples and 56 international samples (see Supplementary Tables S1 and S2). All DNA sequences generated in this study were submitted to GenBank.

### Field Validation of the Franklin three9 System

Validation of the field assay using the Franklin system was conducted on 24 leaf mine samples from multiple plant species collected in Melbourne, Victoria. These included nasturtium (*Tropaeolum majus*), a known host of *L. brassicae*, as well as sow thistle (*Sonchus oleraceus*) and English daisy (*Bellis perennis*), which are common hosts of *P. syngenesiae*.

## Results

### Assay Performance and Specificity

We developed 2 species-specific TaqMan assays targeting: (i) a 129 bp fragment of the mtDNA ND5 region of *L. bryoniae*, and (ii) a 146 bp fragment of the mtDNA CO1 region of *L. chinensis*. Due to *in silico* analysis suggesting that the *L. huidobrensis* assay developed by Sooda et al. (2017) was likely to amplify *L. bryoniae*, we also designed a new assay for *L. huidobrensis* targeting a 132 bp fragment of the mitochondrial ND5 gene region. All primer and probe sequences used in this study are provided in Table 3.

DNA was extracted from 98 samples, and the mtDNA CO1 barcoding region was amplified and sequenced to confirm species identification. Sequence data were successfully obtained from 90 samples, representing all 6 target *Liriomyza* species and 9 non-target species. Of the 8 samples that failed to amplify, 4 were *C. milleri* flies from Australia (Supplementary Table S1) and the remaining 4 were from Kenya (Supplementary Table S2). There was no previous CO1 reference material for *L. katoi*, *L. yasumatsui, O. alysicarpi* or *O. solanicola* on the BOLD database or GenBank. Additionally, CO1 sequence data generated for *O. solanicola* individuals (which we identified morphologically via detailed examination of male genitalia under a microscope) was found to cluster withing a group of *Melanagromyza metallica* CO1 sequences on BOLD with 97% similarity to accession HQ945421. Our CO1 sequence data generated for *O. alysicarpi* (also identified morphologically) matched an unidentified Agromyzinae species on GenBank (accession: KR476577), which was collected from *Alysicarpus ovalifolius* (Blacket et al. 2015).

We then tested the new assays (for *L. bryoniae* and *L. chinensis*, and the revised *L. huidobrensis* assay), alongside the original assays from Sooda et al. (2017) and Pirtle et al. (2021) (targeting *L. sativae*, *L. huidobrensis*, *L. trifolii*, and *L. brassicae*) on 308 Australian samples and 56 samples from other countries (Tables 1 and 2; Supplementary Fig. S1). Target species included: 21 *L. sativae* (from 6 countries), 130 *L. trifolii* (7 countries), 130 *L. huidobrensis* (3 countries), 28 *L. brassicae* (3 countries), 3 *L. bryoniae* (one country), and 3 *L. chinensis* (one country). All newly developed assays amplified only their intended target species at low *Cq* values, with no off-target amplification at comparable *Cq* thresholds, indicating strong specificity. The assays for *L. sativae* and *L. trifolii* from Sooda et al. (2017) similarly showed high specificity. In contrast, the original *L. huidobrensis* assay from Sooda et al. (2017) amplified both *L. huidobrensis* and *L. bryoniae* samples at equivalent *Cq* values (Table 2 and Supplementary Table S2), consistent with predictions from *in silico* analysis. For all Australian and international *L. huidobrensis* samples, *Cq* values were highly consistent between the Sooda et al. (2017) assay and the assay developed in this study (Supplementary Tables S1 and S2). The 4 Kenyan samples that did not amplify with CO1 barcoding primers were successfully identified via our qPCR assays: 3 individuals were *L. trifolii* and one was *L. sativae*. Sample L_bra_027 showed amplification for both *L. brassicae* (*Cq *= 20) and *L. huidobrensis* (*Cq *= 32). As flies of both species were collected in the same tube at Mt Whitestone (Queensland), the weaker *L. huidobrensis* signal is very likely due to cross-contamination rather than a true mixed-species result of the assay. The *Cq* values for every sample, including the sample type (fly, pupa, larva, leaf mine) are shown in Supplementary Tables S1 and S2, with leaf mines generally having high *Cq* values and tissue samples (fly, pupa, larva) having lower *Cq* values.

Sequences generated in this study are deposited in GenBank under accession numbers PV988230-PV988283 (*C. humeralis*), PV988242-PV988243 (*L. chenopodii*), PV988244 (*O. alysicarpi*), PV988245 (*O. solanicola*), PV988246-PV988248 (*P. plantaginis*), PV988249-PV988263 (*P. syngenesiae*), PV988264-PV988267 (*S. flava*), PV988268-PV988283 (*L. sativae*), PV988284-PV988298 (*L. trifolii*), PV988299-PV988307 (*L. huidobrensis*), PV988308-PV988310 (*L. bryoniae*), PV988311-PV988313 (*L. chinensis*), PV988239-PV988241 & PV988314-PV9888317 (*L. brassicae*), PV988318 (*L. katoi*), and PV988319 (*L. yasumatsui*).

### Assay Efficiency and Sensitivity

Standard curves were generated from serial dilutions of DNA from each target species for all 7 qPCR assays, as well as the *L. brassicae* assay undertaken on the Franklin system (Table 4; Supplementary Fig. S2). A strong linear relationship was observed between *Cq* values and the log of the starting DNA concentration for all assays (*R*^2^ = 1.0 for the 7 assays undertaken on the Roche LightCycler; *R*^2^ = 0.98 for the *L. brassicae* assay on the Franklin system). Amplification efficiency ranged from 91% to 98%. The *L. sativae* assay demonstrated the highest efficiency (98%), followed by the *L. trifolii*, *L. brassicae*, and the 2 *L. huidobrensis* assays (each 96%). The *L. bryoniae* assay showed the lowest efficiency (91%). The LOD values ranged from 3.54 (*L. trifolii* and *L. brassicae*) to 75.8 fg (original *L. huidobrensis* assay) of gDNA. The LOQ values ranged from 7 (*L. chinensis*) to 949 fg (*L. trifolii*) of gDNA.

**Table 4.** Summary of results from the curve-fitting method of Klymus et al. (2020) used to evaluate primer efficiency, LOD, and LOQ for the species-specific assays used in this study

Assay	*R* ^b^ value	Slope	Intercept	LOD	LOQ	Efficiency value	Efficiency %
***Liriomyza sativae* ^a^**	1.00	−3.38	41.01	5.56	22	1.976	98
***Liriomyza trifolii* ^a^**	1.00	−3.42	39.23	3.54	949	1.963	96
***Liriomyza huidobrensis* ^a^**	1.00	−3.41	40.00	75.80	82	1.965	96
***Liriomyza huidobrensis* ^b^**	1.00	−3.41	38.24	12.34	55	1.965	96
***Liriomyza brassicae* (LightCycler)^c^**	1.00	−3.41	40.60	3.54	17	1.964	96
***Liriomyza brassicae* (Franklin)^c^**	0.98	−3.46	-	-	-	1.945	94
***Liriomyza bryoniae* ^b^**	1.00	−3.57	40.40	31.60	110	1.905	91
***Liriomyza chinensis* ^b^**	1.00	−3.54	38.34	3.60	7	1.917	92

a From assay developed by Sooda et al. (2017).

b From assays developed in this study.

c From assay developed by Pirtle et al. (2021).

### Field Testing Using the Franklin three9 System

We compared the performance of the Franklin system to the Roche LightCycler using a standard dilution series of *L. brassicae* DNA. The assay showed slightly lower efficiency on the Franklin (94%) compared with the LightCycler (96%), however the average *Cq* values across the 5 dilutions was very similar (29.74 Franklin vs 30.39 Roche). Furthermore, the *Cq* values were tightly correlated between the 2 assays (*r *= 0.99).

We then tested fresh *L. brassicae* larvae excised from leaf mines in a laboratory colony using the Franklin system and the rapid DNA extraction protocol. All 8 samples produced positive results, with an average *Cq* value of 22.91, while the negative control returned no amplification. We also tested 16 *Brassica rapa* subsp. *chinensis* empty leaf mines from the laboratory colony, all of which produced positive results with an average *Cq* value of 34.73, while the 2 negative controls showed no amplification. To test naturally occurring samples in the field, we collected empty leaf mines from 3 host plants. Leaf mines from *T. majus* yielded 6 out of 8 positive detections for *L. brassicae*, with an average *Cq* value of 34.88; the 2 negative samples showed evidence of PCR inhibition. All leaf mines collected from *S. oleraceus* and *B. perennis* were qPCR-negative and showed high levels of inhibition, which likely affected detection. Based on known plant host associations, the detection of *L. brassicae* in *T. majus* was expected, while *S. oleraceus* and *B. perenni*s were most likely mined by *P. syngenesiae*, with negative results expected in the absence of inhibition.

## Discussion

This study provides 2 major advances that strengthen molecular diagnostics for *Liriomyza* pest species of biosecurity concern in Australia. First, we developed and validated species-specific qPCR assays for *L. bryoniae* and *L. chinensis*, and revised the existing *L. huidobrensis* assay to overcome cross-reactivity with *L. bryoniae*. Second, we demonstrated the applicability of these assays in a portable, field-ready format using the Franklin system. Importantly, the workflow was validated on both laboratory-reared and field-collected samples, with the entire process, from sample collection to result, taking ∼90 min. The Franklin system could be utilized in a variety of biosecurity settings, having already been applied successfully in such contexts, with examples in other species including the detection of Khapra beetle, *Trogoderma granarium* Everts (Trujillo‑González et al. 2022) and the Siberian silk moth *Dendrolimus sibiricus* Tschetverikov (Stewart et al. 2023).

Our newly developed assays exhibited strong specificity, with no cross-amplification detected among 16 *Liriomyza* and other agromyzid species spanning a broad geographic range. By testing specimens collected from multiple locations and countries, we confirmed that the assays for *L. sativae, L. trifolii, L. bryoniae, L. chinensis*, and *L. brassicae* were highly specific to their intended targets. In contrast, the *L. huidobrensis* assay of Sooda et al. (2017), which targets the mtDNA CO1 region, consistently cross-amplified *L. bryoniae* at similar *Cq* values, limiting its diagnostic reliability in regions where both species may co-occur. To resolve this, we designed a new TaqMan assay targeting the mitochondrial ND5 region, identified through *in silico* analysis as being diagnostic for *L. huidobrensis*. This revised assay eliminated the cross-reactivity issue while maintaining high specificity across tested species. Although sample numbers for *L. bryoniae* and *L. chinensis* were limited, specificity was robust within the available dataset. Importantly, the sensitivity of all assays remained high across a broad range of DNA concentrations, with limits of detection ranging from 3.54 to 75.8 fg, and amplification efficiencies exceeding 90%. Collectively, these tools represent a significant advance for the surveillance of *L. sativae, L. trifolii, L. huidobrensis, L. bryoniae*, and *L. chinensis*—all species of biosecurity concern in Australia. While the first 3 are now established (Blacket et al. 2015, IPCC 2017, 2021a, 2021b, Mulholland et al. 2022), their current distributions remain restricted relative to predicted ranges (Maino et al. 2023).

We have demonstrated the practical utility of these assays in an operational context. Following the first detection of *L. huidobrensis* in Victoria (Australia) in 2022, we received multiple celery samples from supermarkets in Melbourne (Victoria) showing evidence of leaf mining. Using the newly developed assays, we were able to extract DNA from empty leaf mines and confirm the presence of *L. huidobrensis*, marking further detections of this pest. In response to industry concern, we subsequently established a grower-facing diagnostic service, distributing a standardized sampling protocol to relevant stakeholders. Between October and December 2022, Victorian growers submitted 24 leaf mine samples for testing, which were analyzed using the multi-species qPCR assays. Results were returned to growers within 3 days of sample receipt. All samples tested negative for *L. trifolii*, *L. huidobrensis*, *L. sativae*, *L. brassicae*, *L. bryoniae*, and *L. chinensis*. Where DNA quality allowed, negative qPCR results were validated via CO1 sequencing, which identified the leafminers as *S. flava*. This workflow highlights the speed, accessibility and reliability of the assays under real-world conditions, and their capacity to deliver high-confidence results for industry stakeholders during potential incursion events.

Our assays add to the expanding suite of molecular tools available for *Liriomyza* diagnostics, reflecting a broader shift towards more sensitive, specific, and field-deployable surveillance methods. For example, Zhu et al. (2023) recently developed Loop-mediated isothermal amplification (LAMP) assays for *L. huidobrensis* targeting both mitochondrial and nuclear markers to enable rapid in-field detection. These highly specific single-species assays complement our multi-species qPCR panel, which can simultaneously identify all 6 key *Liriomyza* pests. Similarly, Sooda et al. (2017) improved diagnostic efficiency by combining assays for *L. sativae*, *L. trifolii*, and *L. huidobrensis* into a multiplex real-time assay. In the present study, we extended this approach by designing new assays for additional species. Users could combine these assays into multiplex qPCR reactions to suit their platform’s detection capabilities and surveillance priorities, increasing flexibility for high-throughput screening or targeted diagnostics. However, combinations should be tested with the intended platform and target species to ensure assay performance is maintained. Another important advancement has been the extension of eDNA methods, originally developed for *L. sativae* (Pirtle et al. 2021), to enable detection of a range of *Liriomyza* spp. from empty leaf mines. Collectively, these developments illustrate the increasing utility of molecular diagnostics in overcoming the challenges of identifying morphologically similar agromyzids, particularly when specimens are degraded, immature, or absent. It will be important that these molecular tools are incorporated into existing international and national diagnostic protocols for *Liriomyza* spp. (IPPC 2016, Malipatil et al. 2016).

While the Qiagen Buffer AE extraction method enabled rapid field testing and produced consistent results for *L. brassicae* larvae, it was less effective for screening empty leaf mines. This likely reflects the variable age of leaf mines and the degraded, low-quantity DNA they often contain (Derocles et al. 2015). To improve performance under diverse field conditions and across host plants with different tissue characteristics, alternative extraction protocols and/or further optimization will be required. In particular, samples from plants with thicker or tougher leaves, or those stored under suboptimal conditions, may yield lower-quality DNA and reduce detection sensitivity (Zhu et al. 2024). Future work could also evaluate the inclusion of internal positive controls to detect PCR inhibition and explore preservation methods better suited to field collection and passive surveillance. Preliminary testing on the Franklin system showed that *L. brassicae* larvae excised from leaf mines and processed directly in Qiagen Buffer AE yielded strong *Cq* values, whereas empty leaf mines produced inconsistent results. The rapid DNA extraction method will likely require modification to overcome inhibitors commonly present in plant tissues, such as polysaccharides and phenolic compounds (Jobes et al. 1995). Sample size was another limitation of our study, with relatively few individuals tested for some species, most notably *L. bryoniae* and *L. chinensis*, as well as several non-target agromyzids. While results for these species were internally consistent, broader testing across additional populations and geographic ranges is needed to fully confirm assay robustness.

This study also contributes to filling important taxonomic and genetic gaps in publicly available databases. Through CO1 barcoding, we generated the first published sequences for several agromyzid species, including *L. katoi*, *L. yasumatsui*, *O. alysicarpi*, and *O. solanicola*, none of which were previously represented in GenBank or the BOLD database. We also resolved the identity of an ambiguous Agromyzinae sp. sequence in GenBank (accession: KR476577) reported by Blacket et al. (2015); originally collected from *A. ovalifolius* in Weipa (Queensland), this sequence was found to be a 100% match with *O. alysicarpi* individuals collected in this study from Lakeland (Queensland). Our sequence data for *O. solanicola* was obtained from flies collected from eggplant (*Solanum melongena*) in Queensland, with species identity confirmed via male genitalia, following Spencer (1977). The closest match with 97% sequence similarity was *M. metallica* (accession: HQ945421), a specimen that was identified by an experienced citizen scientist, but lacking expert taxonomic verification. In Australia, *M. metallica* has only been recorded from *Ageratum conyzoides* and *Bidens pilosa* (both Asteraceae), with larvae feeding and pupating in the stems of plants (Spencer 1977). Further targeted sampling and sequencing of these species within the recently established Ophiomyiinae subfamily (Xuan et al. 2023) are needed to ensure accurate reference data, supported by voucher specimens lodged in museum collections (Lue et al. 2021). Although 2 adult *L. chenopodii* were collected from *T. majus*, no larvae were observed in the leaves, and, therefore, we do not regard this as evidence of a new host association. This is consistent with Spencer (1977), who reported host plants of *L. chenopodii* to be restricted to species within Caryophyllaceae and Chenopodiaceae. Collectively, these findings highlight the importance of integrating molecular screening with careful morphological identifications, particularly for groups such as Agromyzidae where sequence reference libraries remain incomplete (Lue et al. 2021).

In conclusion, this study provides new molecular tools for the accurate, rapid, and field-deployable identification of *Liriomyza* pests of concern in Australia. These assays are already being integrated into biosecurity and surveillance workflows, with demonstrated value for both incursion detection and grower-facing diagnostics. As *Liriomyza* incursions continue to emerge across the country, such diagnostic tools will facilitate early detection, supporting rapid response, and underpinning effective pest management.

## Supplementary Material

toag119_Supplementary_Data
