# Supplementary material for: New species-specific quantitative PCR assays for Liriomyza leafminers: supporting biosecurity and in-field identification
Source: J Econ Entomol. 2026 May 11;119(3):2295–305. doi: 10.1093/jee/toag119 (PMC13268535; doi:10.1093/jee/toag119)
Supplement: toag119_Supplementary_Data [file toag119_supplementary_data.zip › vanRooyen et al_Suppl Figures_18 Feb 2026.docx]

**qPCR curve for: Liriomyza sativae 1**


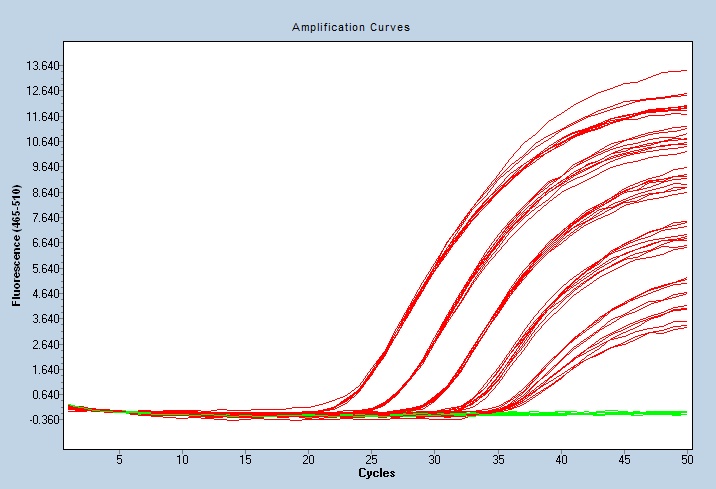


**qPCR curve for: Liriomyza trifolii 1**
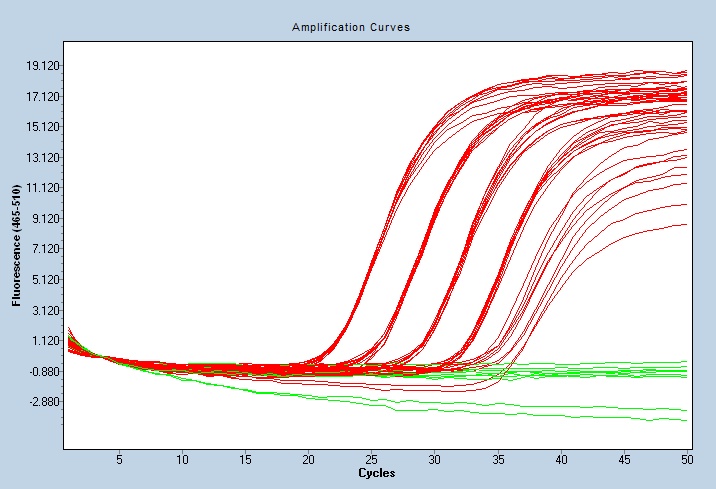


**qPCR curve for: Liriomyza huidobrensis 1**


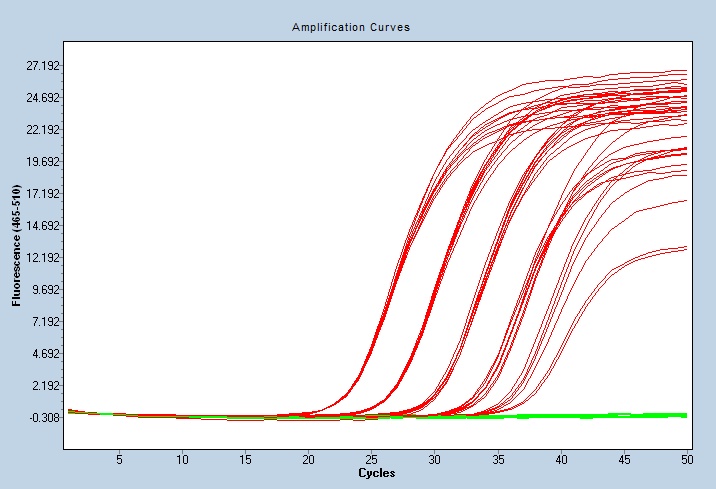


**qPCR curve for: Liriomyza huidobrensis 2**
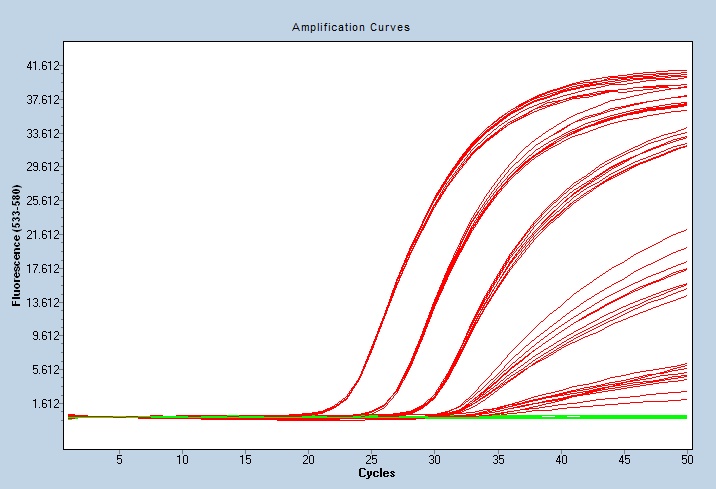


**qPCR curve for: Liriomyza brassicae 3**
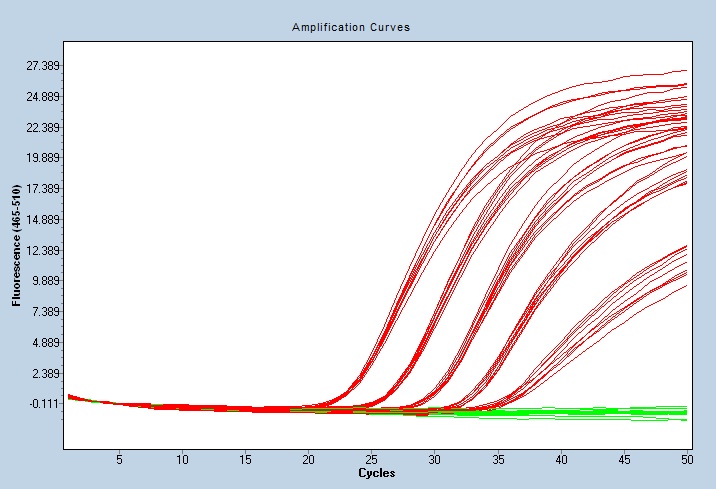


**qPCR curve for: Liriomyza bryoniae 2**
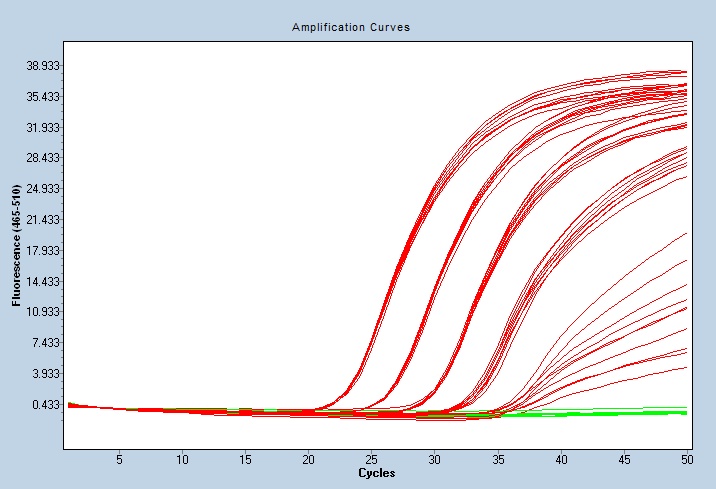


**qPCR curve for: Liriomyza chinensis** **2**
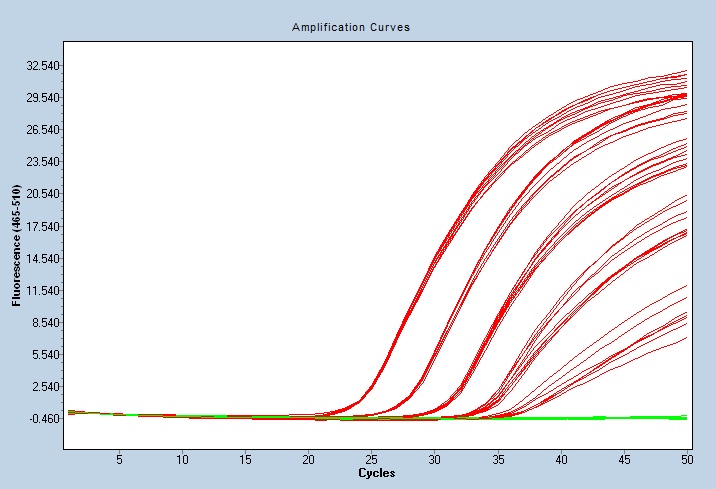


**Suppl. Figure 1. Quantitative PCR (qPCR) curves for the species-specific assays used in this study.** 1 = from assay developed by Sooda et al. (2017). 2 = from assays developed in this study. 3 = from assay developed by Pirtle et al. (2021).

**Suppl. Figure 2. Results from the curve-fitting method of Klymus et al. (2020) used to evaluate primer efficiency, limit of detection (LOD), and limit of quantification (LOQ) for the species-specific assays used in this study.** 1 = from assay developed by Sooda et al. (2017). 2 = from assays developed in this study. 3 = from assay developed by Pirtle et al. (2021).

**References**

Klymus KE, Merkes CM, Allison MJ, et al. 2020. Reporting the limits of detection and quantification for environmental DNA assays. Environmental DNA, 2, 271–282. https://doi.org/10.1002/edn3.29

Pirtle EI, van Rooyen AR, Maino J, et al. 2021. A molecular method for biomonitoring of an exotic plant-pest: Leafmining for environmental DNA. Mol. Ecol. 30:4913–4925. https://doi.org/10.1111/mec.16092

Sooda A, Gunawardana D, Li D, et al. 2017. Multiplex real-time PCR assay for the detection of three invasive leafminer species: *Liriomyza huidobrensis*, *L. sativae* and *L. trifolii* (Diptera: Agromyzidae). Austral Entomol. 56:153-159 https://doi.org/10.1111/aen.12237
